# Unraveling Nonketotic Hyperglycemia Hemichorea-Hemiballismus Syndrome: A Case Report of Diagnosis and Management

**DOI:** 10.7759/cureus.65094

**Published:** 2024-07-22

**Authors:** Charuta Gadkari, Waseem M Ilyas, Aditya Pundkar, Rajshree Devi Seram, Preethy Koshy, Aniket Patel

**Affiliations:** 1 Emergency Medicine, Jawaharlal Nehru Medical College, Datta Meghe Institute of Higher Education and Research, Wardha, IND; 2 Orthopedics, Jawaharlal Nehru Medical College, Datta Meghe Institute of Higher Education and Research, Wardha, IND

**Keywords:** chorea and hemiballismus, diabetic hemi-chorea, hemichorea-hemiballismus, type-2 diabetes mellitus, nonketotic hyperglycemia

## Abstract

Nonketotic hyperglycemia hemichorea-hemiballismus syndrome (NHH) is an uncommon neurological condition linked to poorly managed diabetes mellitus (DM). It presents with spontaneous, erratic movements that impact just one side of the body. Our case of NHH was of a 76-year-old female with uncontrolled type 2 DM, ischemic heart disease, and dilated cardiomyopathy. Despite previous treatment for similar symptoms, the patient developed left-sided choreo-ballistic movements. Despite difficulties obtaining clear magnetic resonance imaging (MRI) due to involuntary movements, the image revealed T1 hyperintense signals in the right lentiform nucleus and subtle signals in the left lentiform nucleus and external capsule. Management included insulin, tetrabenazine, haloperidol, lorazepam, and other adjunctive therapies, resulting in symptom resolution by the fourth day. This case underscores the importance of considering NHH in patients with uncontrolled DM presenting with abnormal movements, highlighting the challenges in imaging due to involuntary movements and emphasizing the need for aggressive glycemic control and treatment strategies.

## Introduction

Nonketotic hyperglycemia hemichorea-hemiballismus syndrome (NHH), alternatively termed *diabetic striatopathy* (DS) or *nonketotic hyperglycemic hemichorea* or *chorea, hyperglycemia, basal ganglia (C-H-BG) syndrome*, is a rare neurological condition associated with nonketotic hyperglycemia. It is most commonly seen in elderly females [1-3]. NHH is associated with uncontrolled diabetes mellitus (DM), characterized by involuntary choreo-ballistic movements predominantly affecting one side of the body in the absence of ketoacidosis. The pathophysiology of NHH involves alterations in glucose metabolism leading to neuronal dysfunction in the basal ganglia, particularly the striatum [1-4]. The prevalence of NHH is approximately one in 100,000. However, it is very likely to be higher as there is a lack of awareness among physicians. Among patients with poorly controlled type 2 diabetes, around 0.58% have NHH. This percentage increases to 1.2% among those hospitalized specifically for neurological symptoms [3].

NHH can appear either as the initial presentation of undiagnosed DM or as a complication in individuals already diagnosed with diabetes. Other than nonketotic hyperglycemic hemichorea (NHH), the hemichorea-hemiballismus syndrome has also been observed in cases of stroke, brain tumors, vascular malformation, vasculitis, demyelination disorders, infections such as tuberculoma and cerebral toxoplasmosis, and trauma [5]. Diagnosis of NHH relies on clinical recognition, as typical neuroradiological findings may be absent in a significant proportion of cases. Management of NHH involves aggressive control of hyperglycemia, often resulting in the resolution of symptoms [1,2,6]. We present a case of NHH in a 76-year-old female with a history of poorly controlled type 2 DM, ischemic heart disease, and dilated cardiomyopathy. Despite previous treatment for similar symptoms, the patient developed left-sided choreo-ballistic movements. This case highlights the importance of prompt recognition and management strategies in patients with NHH.

## Case presentation

A 76-year-old female with a history of uncontrolled type 2 DM, hypertension, ischemic heart disease, and dilated cardiomyopathy presented to the emergency department with abnormal movements on the left side of her body. Her relatives reported three days of intermittent, irregular movements involving the left arm and leg, without any associated fever, altered sensorium, seizures, or involuntary passage of stools/urine. On examination, the patient was drowsy but responsive, with elevated bedside blood sugar levels (232 mg/dL). Vitals were normal. A neurological examination revealed irregular, flinging movements of the left arm and leg, which the patient could not voluntarily stop (Video 1). Muscle tone and power were normal, and she could sit with mild support. Sensory examination and cranial nerve testing were unremarkable.

**Video 1:** Involuntary choreo-ballistic movements affecting the left side of the body.

**Figure 1:**
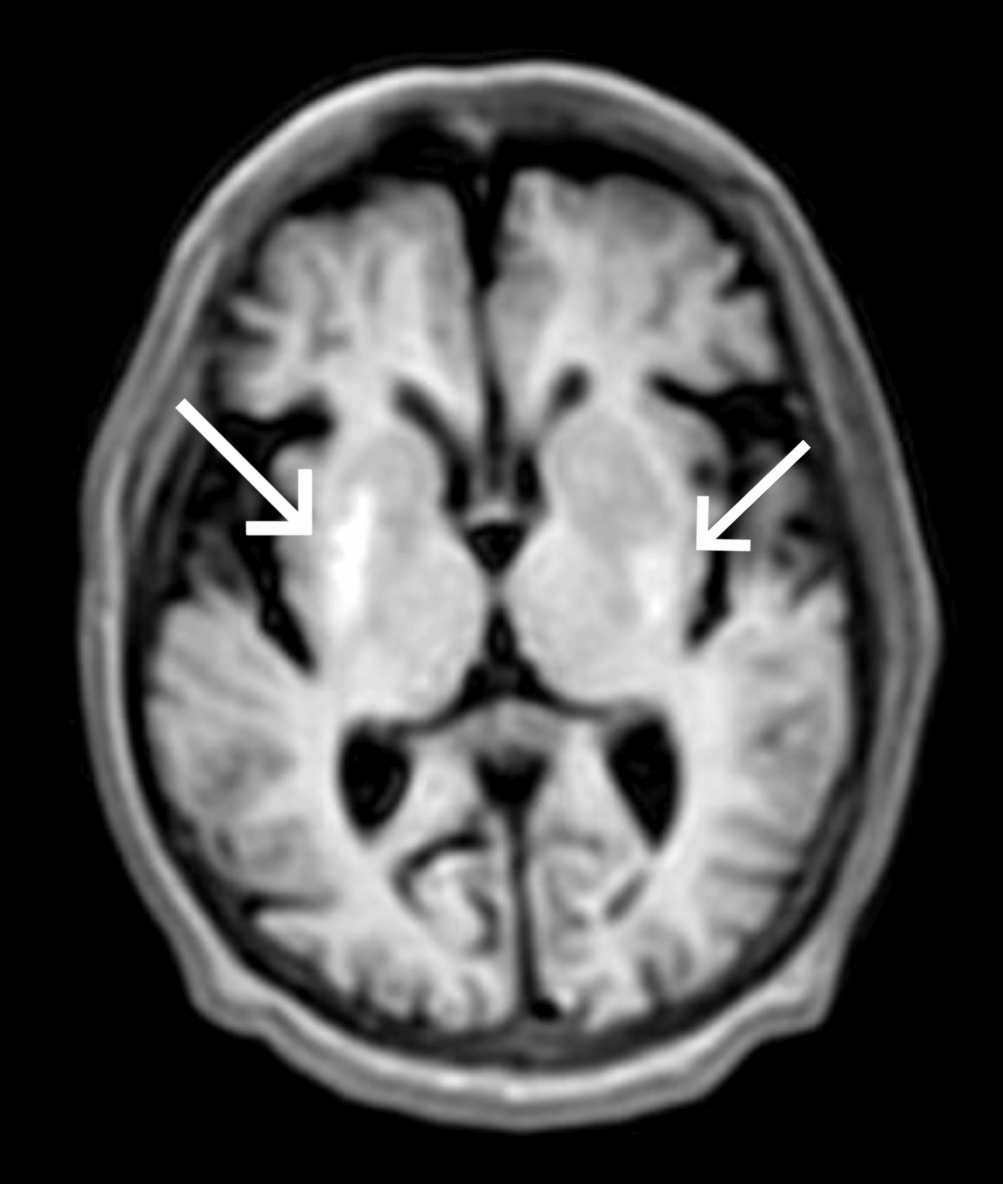
T1 hyperintense signal in the right lentiform nucleus and subtle T1 hyperintense signal in the left lentiform nucleus and external capsule. Arrows indicate the lesions. The image quality is slightly compromised due to involuntary movements of the patient during the scan, resulting in slight blurriness.

Obtaining a clear magnetic resonance imaging (MRI) brain scan was challenging due to the patient's choreo-ballistic movements. Mild sedation was administered with haloperidol 5 mg and promethazine 25 mg to facilitate the imaging process; however, the patient moved during the scan, resulting in a slightly blurry image. Deep sedation was not an option as it could have threatened the airway. Despite the slight blurriness, the radiologist present during the scan confirmed that the image quality was sufficient to visualize the pathology. The MRI revealed T1 hyperintense signals in the right lentiform nucleus, which were consistent with acute findings, and subtle T1 hyperintense signals in the left lentiform nucleus and external capsule, suggesting chronic changes (Figure 1). These findings and the clinical presentation supported a diagnosis of NHH. Four months prior, the patient had a similar episode involving involuntary movements of the right upper limb and tongue, accompanied by tingling sensations in the palms and soles. This previous episode was also diagnosed as nonketotic hyperglycemic hemichorea and treated accordingly.

The patient received a comprehensive treatment regimen in the intensive care unit (ICU). On the first day of admission, she was administered tetrabenazine 25 mg twice daily, vitamin B complex supplementation, antacids, and her routine combination of rosuvastatin 20 mg and aspirin 75 mg. Despite this, the choreo-ballistic movements persisted, prompting the addition of escitalopram 5 mg and clonazepam 0.5 mg at night, along with lorazepam 0.5 mg in the morning on the second day. The cardiologist also introduced a combination of valsartan 26 mg and sacubitril 24 mg, given as half a tablet twice daily.

2D echocardiography revealed mild left ventricular hypertrophy with grade 1 diastolic dysfunction. The patient's glycosylated hemoglobin (HbA1C) level was 14.5%, indicating uncontrolled DM. Other lab values, including blood counts, liver and renal function tests, serum pH, and serum electrolytes, were normal, and urine ketones were negative. Glucose levels were closely monitored and controlled with insulin therapy during the hospital stay.

As the movements continued into the third day, haloperidol 2 mg twice daily was added to the regimen. By the fourth day, the choreo-ballistic movements had subsided, and the same treatment was maintained. On the fifth day, the combination of escitalopram and clonazepam, as well as lorazepam, was discontinued, while the other medications were continued for an additional four days.

After her symptoms improved, the patient remained in the hospital for observation and further management. Upon discharge, she was advised to continue tetrabenazine 25 mg once daily, haloperidol 2 mg twice daily, the valsartan and sacubitril combination (half a tablet twice daily), rosuvastatin 20 mg and aspirin 75 mg combination tablet once daily, insulin therapy for glycemic control, antacids, and multivitamins for two weeks. She was also advised to review with a neurologist after two weeks and to follow up regularly with her healthcare provider for further management of her DM and associated comorbidities.

## Discussion

NHH is a debilitating neurological complication of uncontrolled DM. NHH poses a diagnostic challenge owing to its rarity and the absence of a consistent pathognomonic neuroradiological finding, and hence, its diagnosis relies mainly on clinical recognition. NHH presents with involuntary, irregular movements affecting one side of the body, often in the absence of diabetic ketoacidosis. The pathophysiology involves alterations in glucose metabolism that lead to neuronal dysfunction in the basal ganglia, particularly the striatum [1-4]. Our patient presented with left-sided choreo-ballistic movements consistent with NHH. Despite prior treatment for similar symptoms, the patient experienced recurrence necessitating hospitalization, underscoring the chronic and multifactorial nature.

Management strategies in our patient included aggressive control of hyperglycemia, with insulin therapy as the mainstay. As per previous studies, most patients with NHH recover with insulin therapy alone. Optimizing glycemic control not only alleviates acute symptoms but also reduces the risk of long-term complications associated with uncontrolled DM [1-3]. Glycosylated hemoglobin levels are very high in most patients, often reaching levels as high as 14%, as was the case in our patients, suggesting that diabetes was uncontrolled [4,7,8]. Adjunctive medications such as haloperidol, tetrabenazine, risperidone, and tiapride have been documented to be effective in alleviating choreo-ballistic movements. Benzodiazepines such as lorazepam and clonazepam are employed to alleviate anxiety, promote relaxation, and reduce the intensity of involuntary movements associated with NHH. Selective serotonin reuptake inhibitors, like escitalopram oxalate, are also an option to address the hyperkinetic movements in NHH [9-11]. Our patient had uncontrolled diabetes, but during the hospital stay, glucose levels were kept within normal limits, and tetrabenazine, haloperidol, lorazepam, and escitalopram oxalate were used to alleviate the hyperkinetic movements. For refractory cases of NHH, surgical treatments like ventrolateral thalamotomy, globus pallidus internus deep brain stimulation, pallidotomy, and transcranial magnetic stimulation have been tried [6,9]. Most patients with NHH have complete symptom relief in seven days - categorized as *early responders* and *late responders* take more than seven days to respond to treatment. One study revealed that 47.5% of patients experienced early and complete alleviation of symptoms, while 28.8% exhibited a delayed response but achieved complete reversal, with 23.7% of cases showing partial recovery [6]. Our patient’s hyperkinetic movements subsided only after four days, which puts her in the category of an *early responder*.

A study on patients with choreo-ballistic movements found that 5% to 45% of clinically isolated DS cases had no neuroimaging findings. In another study encompassing both choreo-ballistic and non-choreo-ballistic movements, it was discovered that only 44% of cases exhibited alterations in brain MRI [6]. So, although MRI may show typical changes in the basal ganglia, its utility may be limited. Hence, a thorough clinical evaluation is the mainstay for diagnosing and managing NHH [6,10]. Lesions commonly appear in the globus pallidus and putamen, detectable through computed tomography (CT) or MRI. In the initial stages, CT of the brain may not show any abnormality, but later, hyperdensity in the striatal region (caudate nuclei and putamen) may become evident. However, MRI of the brain is the imaging modality of choice and shows signal changes in the same region. MRI scans typically reveal specific characteristics in NHH cases, including hyperintensity on T1, variable but hypointense signals on T2/fluid-attenuated inversion recovery (FLAIR) imaging, increased susceptibility on susceptibility-weighted imaging (SWI), and high diffusion signal on diffusion-weighted imaging (DWI). The most consistent finding of the disease is generally the T1 hyperintensity. If the observed imaging findings are unilateral, then usually, the ballistic and choreiform movements correspond with the contralateral side [1,6]. Our patient had a T1 hyperintense signal in the right lentiform nucleus and had chorea-hemiballismus movements on the left side, which is as per the recorded literature. However, our patient also had a subtle T1 hyperintense signal in the left lentiform nucleus and external capsule, which could probably be the remnant lesion from the previous episode of NHH the patient suffered four months back when she had hyperkinetic movements of the right side and tongue.

Despite initial symptom resolution and successful management during hospitalization, recurrence of NHH remains a concern. Nearly 20% of cases may clinically recur, underscoring the chronic and multifactorial nature of the syndrome. This highlights the importance of long-term follow-up and vigilance in monitoring for symptom recurrence, necessitating comprehensive and individualized management strategies tailored to each patient’s specific clinical presentation and underlying comorbidities. Ongoing optimization of glycemic control and close monitoring for potential adverse effects and drug interactions are essential to mitigate the risk of symptom relapse and improve long-term outcomes in patients with NHH [1-4,6].

## Conclusions

Our case underscores the importance of considering NHH in patients presenting with involuntary movements, particularly in the setting of uncontrolled diabetes, the importance of clinically diagnosing NHH, and the role of MRI brain scan and highlights the need for glycemic control to prevent its recurrence and multidisciplinary management approach. Good clinical recognition skills and multimodal treatment strategies are crucial for symptom resolution in patients with NHH and for preventing long-term complications. Effective management involves glycemic control and symptomatic treatment, leading to improved clinical outcomes and quality of life. Further research is warranted to explore optimal treatment strategies and long-term prognosis in patients with NHH.
